# Ablation of the p16^INK4a^ tumour suppressor reverses ageing phenotypes of *klotho* mice

**DOI:** 10.1038/ncomms8035

**Published:** 2015-04-29

**Authors:** Seidai Sato, Yuka Kawamata, Akiko Takahashi, Yoshinori Imai, Aki Hanyu, Atsushi Okuma, Masaki Takasugi, Kimi Yamakoshi, Hiroyuki Sorimachi, Hiroaki Kanda, Yuichi Ishikawa, Saburo Sone, Yasuhiko Nishioka, Naoko Ohtani, Eiji Hara

**Affiliations:** 1 Division of Cancer Biology, Cancer Institute, Japanese Foundation for Cancer Research, Koto-ku, Tokyo 135-8550, Japan; 2 Department of Respiratory Medicine and Rheumatology, University of Tokushima Graduate School of Medicine, Tokushima 770-8503, Japan; 3 CREST, Japan Science and Technology Agency, Kawaguchi, Saitama 332-0012, Japan; 4 Department of Applied Biological Science, Faculty of Science and Technology, Tokyo University of Science, Noda, Chiba 278-8510, Japan; 5 Tokyo Metropolitan Institute of Medical Science, Setagaya-ku, Tokyo 156-8506, Japan; 6 Division of Pathology, Cancer Institute, Japanese Foundation for Cancer Research, Koto-ku, Tokyo 135-8550, Japan; 7 PRESTO, Japan Science and Technology Agency, Kawaguchi, Saitama 332-0012, Japan; 8 Department of Molecular Microbiology, Research Institute for Microbial Diseases, Osaka University, Suita, Osaka 565-0871, Japan

## Abstract

The p16^INK4a^ tumour suppressor has an established role in the implementation of cellular senescence in stem/progenitor cells, which is thought to contribute to organismal ageing. However, since *p16*^*INK4a*^ knockout mice die prematurely from cancer, whether *p16*^*INK4a*^ reduces longevity remains unclear. Here we show that, in mutant mice homozygous for a hypomorphic allele of the *α-klotho* ageing-suppressor gene (*kl*^*kl/kl*^), accelerated ageing phenotypes are rescued by *p16*^*INK4a*^ ablation. Surprisingly, this is due to the restoration of *α-klotho* expression in *kl*^*kl/kl*^ mice and does not occur when *p16*^*INK4a*^ is ablated in *α-klotho* knockout mice (*kl*^*−/−*^), suggesting that p16^INK4a^ is an upstream regulator of *α-klotho* expression. Indeed, p16^INK4a^ represses *α-klotho* promoter activity by blocking the functions of E2Fs. These results, together with the observation that the expression levels of *p16*^*INK4a*^ are inversely correlated with those of *α-klotho* throughout ageing, indicate that *p16*^*INK4a*^ plays a previously unrecognized role in downregulating *α-klotho* expression during ageing.

## Abstract (web summary)

The protein p16^INK4a^ promotes senescence in tissue stem cells and thereby contributes to organismal ageing. Here the authors reveal that p16^INK4a^ also downregulates expression of *a-klotho*, thereby revealing an additional ageing-promoting function of 16^INK4a^ that is independent from its role in senescence.

## Graphical abstract

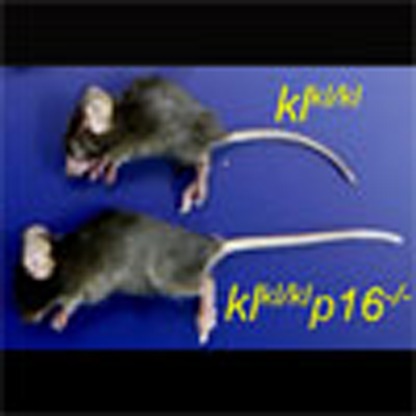

Tissue repair and regeneration are essential for longevity in complex animals, and often depend on the proliferative activity of stem or progenitor cells^1^. In many tissues, the proliferative activity of such cells declines with age, contributing to many ageing-associated pathologies^2^,^3^,^4^. In mammals, the *p16*^*INK4a*^ tumour-suppressor gene elicits irreversible cell-cycle arrest known as cellular senescence^5^,^6^,^7^,^8^,^9^,^10^, and its expression increases with age in many tissues^11^,^12^,^13^, along with the accumulation of dysfunctional senescent stem/progenitor cells^14^,^15^,^16^. However, recent studies using middle-aged mice lacking *p16*^*INK4a*^ (*p16*^*−/−*^ mice) revealed that the ageing-associated induction of *p16*^*INK4a*^ expression reduces the proliferative and regenerative capacities of certain stem/progenitor cells during the ageing process^14^,^15^,^16^. These findings have led to speculation that the induction of *p16*^*INK4a*^ expression and the consequent cellular senescence are causally implicated in ageing-associated declines in stem/progenitor cell functions, thereby reducing longevity. However, since *p16*^*−/−*^ mice die of cancer long before they reach the age at which most normal mice start to die^17^, it remains unclear whether *p16*^*INK4a*^ truly limits longevity in mammals. One approach to circumvent this problem would be the use of short-lived mouse strains with accelerated-ageing phenotypes. However, attempts towards extending the maximum lifespan of accelerated-ageing mouse strains by *p16*^*INK4a*^ ablation have so far been unsuccessful^18^,^19^, raising the question of whether *p16*^*INK4a*^ truly limits longevity in mammals.

Mutant mice homozygous for a severely downregulated hypomorphic allele of the *α-klotho* gene (referred to as *kl*^*kl/kl*^ or *klotho* mice) manifest multiple age-related disorders that are also observed in humans, including infertility, growth retardation, osteoporosis, pulmonary emphysema, skin atrophy, ectopic calcification and shortened lifespan^20^. Furthermore, the levels of *α-klotho* expression decline with age in both humans and mice^21^, and overexpression of *α-klotho* extends the maximum lifespan in mice^22^, suggesting that *α-klotho* acts as an ageing-suppressor gene in mammals^23^. The *α-klotho* gene encodes a single-pass transmembrane protein that is predominantly expressed in the kidney^20^, and to a lesser extent in the brain^24^. Two forms of the *α*-klotho protein exist: a membrane-bound form and a secreted form^25^, and each has different functions^22^,^24^,^26^,^27^. As increases in senescent progenitor cells and decreases in stem cell numbers were observed in several tissues in *kl*^*kl/kl*^ mice^28^,^29^, we wondered whether *p16*^*INK4a*^ contributes to the accelerated-ageing phenotypes in *kl*^*kl/kl*^ mice, by eliciting cellular senescence in certain stem/progenitor cells.

In the present study, we explore the roles of *p16*^*INK4a*^ in accelerated-ageing phenotypes of *klotho* mice. We show that ablation of the *p16*^*INK4a*^ gene reverses various ageing phenotypes, including maximum lifespan, of *kl*^*kl/kl*^ mice. Surprisingly, however, this is due to the restoration of *α-klotho* expression in *kl*^*kl/kl*^ mice and does not occur when *p16*^*INK4a*^ is ablated in knockout mice lacking *α-klotho* (*kl*^*−/−*^), indicating that p16^INK4a^ is an upstream regulator of *α-klotho* expression. Thus, although *p16*^*INK4a*^ has an established role in the implementation of cellular senescence in stem/progenitor cells^5^,^6^,^7^,^8^,^9^,^10^, which are likely to reduce longevity^13^, our results reveal that p16^INK4a^ has an additional function in promoting ageing phenotypes by downregulating *α-klotho* expression in mice. Our findings advance our understanding of the molecular mechanisms underlying the development and progression of ageing in mammals.

## Results

### Ablation of *p16* ^*INK4a*^ reverses ageing phenotypes of *klotho* mice

**Figure 1:**
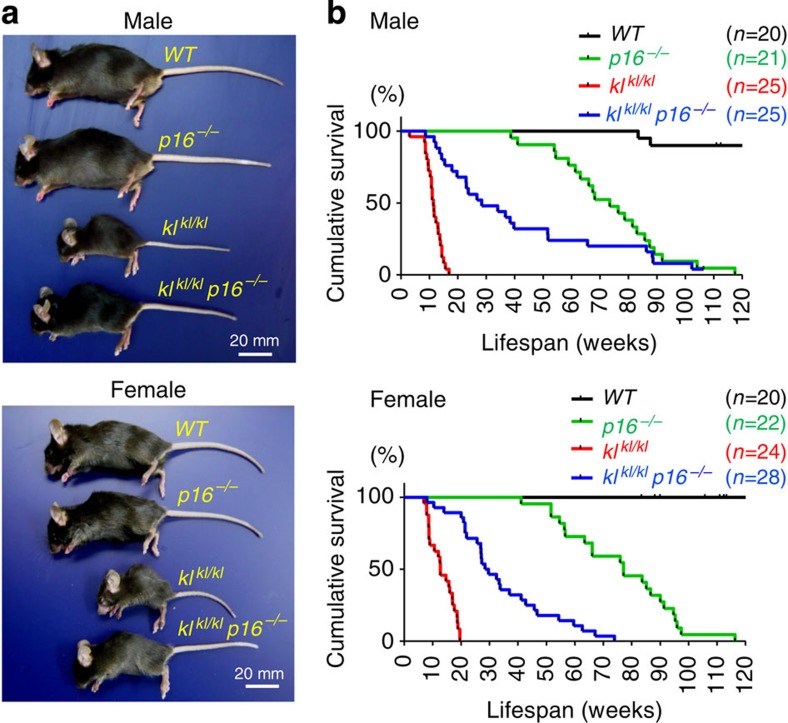
Extension of maximum lifespan of *kl*^*kl/kl*^ mice by *p16*^*INK4a*^ ablation. (**a**) Representative photographs of 11-week-old mice of each genotype (*n*=3). (**b**) Kaplan–Meier plot showing survival of *WT* (male, *n*=20; female, *n*=20)*, p16*^*−/−*^ (male, *n*=21; female, *n*=22)*, kl*^*kl/kl*^ (male, *n*=25; female, *n*=24) and *p16*^*−/−*^ *kl*^*kl/kl*^ (male, *n*=25; female, *n*=28).

**Figure 2:**
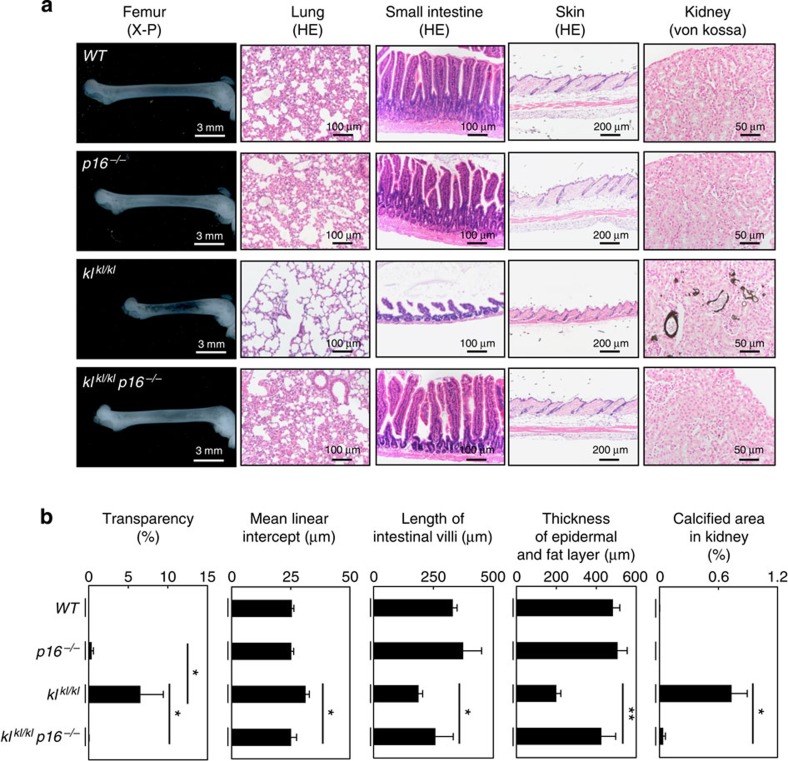
Reversing the ageing phenotypes of *kl*^*kl/kl*^ mice by *p16*^*INK4a*^ ablation. (**a**) Histological analysis of 11-week-old female *WT, p16*^*−/−*^*, kl*^*kl/kl*^ *and p16*^*−/−*^ *kl*^*kl/kl*^ mice. Representative images of bone radiographs of femurs (X-P), HE of tissues indicated top and von Kossa staining (von Kossa) of the kidney for detecting ectopic calcification were shown. (**b**) The histograms indicate the quantitative analysis of X-ray transparency of femur (*WT* (*n*=3)*, p16*^*−/−*^ (*n*=3)*, kl*^*kl/kl*^ (*n*=6) and *p16*^*−/−*^ *kl*^*kl/kl*^ (*n*=3)), the mean linear intercept (Lm) in lung tissue (*WT* (*n*=9)*, p16*^*−/−*^ (*n*=9)*, kl*^*kl/kl*^ (*n*=7) and *p16*^*−/−*^ *kl*^*kl/kl*^ (*n*=9)), intestinal villi length (*WT* (*n*=14)*, p16*^*−/−*^ (*n*=7)*, kl*^*kl/kl*^ (*n*=11) and *p16*^*−/−*^ *kl*^*kl/kl*^ (*n*=3)), epidermal and subcutaneous fat layer thickness (*WT* (*n*=6)*, p16*^*−/−*^ (*n*=6)*, kl*^*kl/kl*^ (*n*=8) and *p16*^*−/−*^ *kl*^*kl/kl*^ (*n*=3)) and the percentages of calcified areas in kidneys (*WT* (*n*=3)*, p16*^*−/−*^ (*n*=3)*, kl*^*kl/kl*^ (*n*=3) and *p16*^*−/−*^ *kl*^*kl/kl*^ (*n*=3)). For graphs of X-ray transparency of femur and Lm in lung tissues, data were analysed by Mann–Whitney *U*-test and are displayed as mean±s.e.m. For graphs of intestinal villi length, epidermal and subcutaneous fat layer thickness and the percentages of calcified areas in kidneys, data were analysed by Student's *t*-test and are displayed as mean±s.e.m. For all graphs: *P<0.05, **P<0.01.

To investigate whether *p16*^*INK4a*^ contributes to the accelerated-ageing phenotypes in *kl*^*kl/kl*^ mice, we first generated *kl*^*kl/kl*^ mice lacking *p16*^*INK4a*^ (*kl*^*kl/kl*^ *p16*^*−/−*^ mice) by cross-breeding heterozygous *klotho* (*kl*^*kl/+*^) mice with heterozygous *p16*^*INK4a*^ knockout (*p16*^*+/−*^) mice, and tested whether the accelerated-ageing phenotypes of *kl*^*kl/kl*^ mice can be reversed by *p16*^*INK4a*^ ablation. Various accelerated-ageing phenotypes of *kl*^*kl/kl*^ mice, such as growth retardation, osteoporosis, pulmonary emphysema and severe atrophy of the intestinal wall and skin, were remarkably mitigated in the *kl*^*kl/kl*^ *p16*^*−/−*^ mice as compared with those in the *kl*^*kl/kl*^ *p16*^*+/*+^ (referred to as *kl*^*kl/kl*^) or *kl*^*kl/kl*^ *p16*^*+/*−^ littermates with the same genetic background (98.375% C57BL/6J, 1.625% C3H/J) (Figs 1 and 2, and data not shown). Moreover, the maximum lifespan of the *kl*^*kl/kl*^ *p16*^*−/−*^ mice was significantly extended as compared with those of the *kl*^*kl/kl*^ or *kl*^*kl/kl*^ *p16*^*+/*−^ littermates, irrespective of gender (Fig. 1b, and data not shown). These results indicated that the accelerated-ageing phenotypes of the *kl*^*kl/kl*^ mice depend strongly on the *p16*^*INK4a*^ status. The simplest explanation for these results would be that the *α*-klotho deficiency caused the ageing phenotypes by elevating *p16*^*INK4a*^ expression, and thus *p16*^*INK4a*^ ablation would mitigate the ageing phenotypes in *kl*^*kl/kl*^ mice.

**Figure 3:**
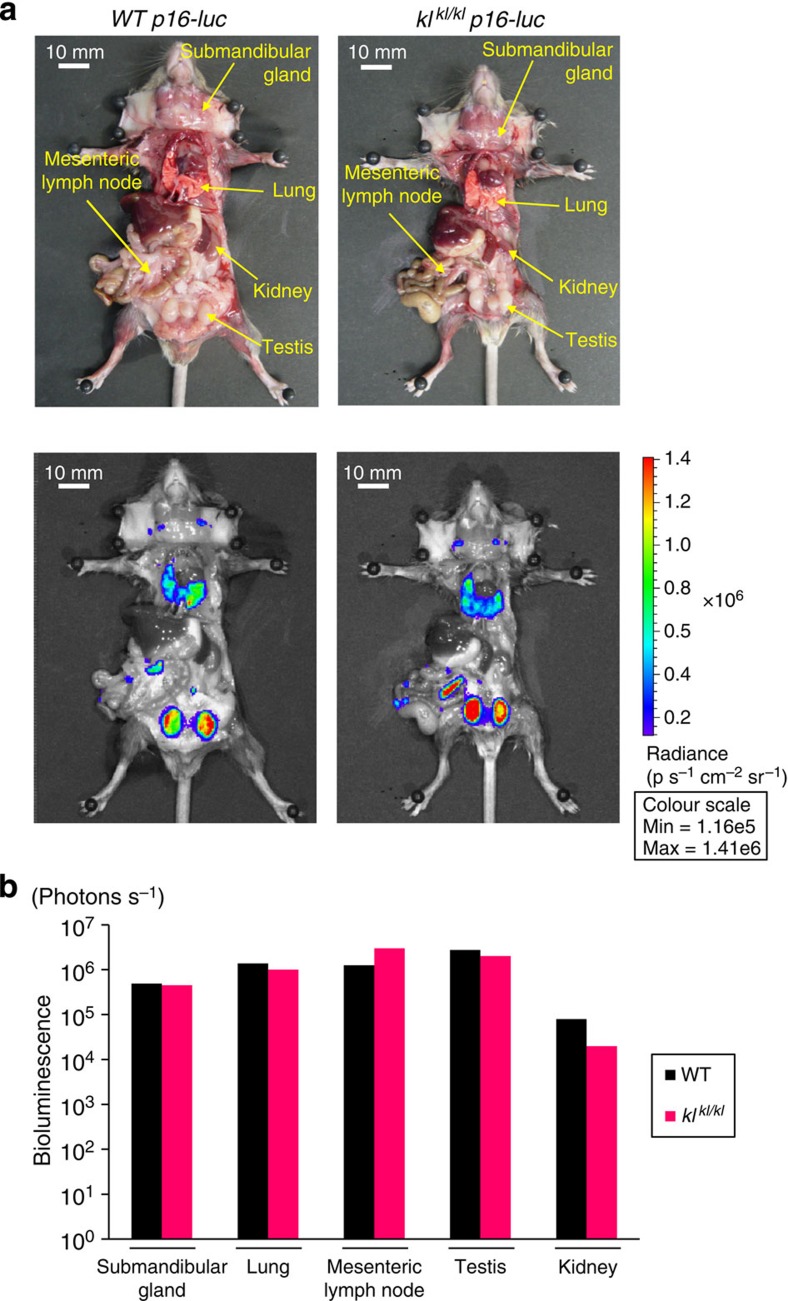
Bioluminescence *in vivo* imaging of *p16*^*INK4a*^ expression in *kl*^*kl/kl*^ mice. (**a**) Littermates of 8-week-old male *p16-luc* mice (*WT p16-luc*) and *p16-luc* mice homozygous for a severely downregulated hypomorphic allele of the *α-klotho* (*kl*^*kl/kl*^ *p16-luc*) were subjected to *in vivo* bioluminescence imaging after being incised through the throat and the anus under anaesthesia. Representative images of three independent experiments are shown (*n*=3 experiments). The colour bar indicates photons with minimum and maximum threshold values. (**b**) Bioluminescence intensity emitted from the organs was graphed (log 10 scale).

To test this hypothesis, we took advantage of using the *p16-luc* mice^12^, in which the levels of *p16*^*INK4a*^ expression can be monitored throughout the body using a bioluminescence imaging (BLI) technique. The *p16-luc* mice were crossed into the *klotho* genetic background and were subjected to BLI. Unexpectedly, however, we were unable to detect any substantial increase of *p16*^*INK4a*^ expression throughout the body in *kl*^*kl/kl*^ mice as judged by BLI (Fig. 3). Because *α-klotho* is predominantly expressed in the kidney^20^,^24^ (see also Supplementary Fig. 1), we further examined the *p16*^*INK4a*^ expression in the kidney using quantitative reverse transcription–PCR (qRT–PCR) analysis. Again, however, there was a slight but insubstantial increase of *p16*^*INK4a*^ expression in the kidneys of *kl*^*kl/kl*^ mice, as compared with *p16*^*INK4a*^ expression in those of wild-type (wt) mice (Supplementary Fig. 2). These results raise a question of how the *p16*^*INK4a*^ ablation reversed the accelerated-ageing phenotypes of *kl*^*kl/kl*^ mice.

### *p16* ^*INK4a*^ ablation restores *α-klotho* expression in *kl* ^*kl/kl*^ mice

**Figure 4:**
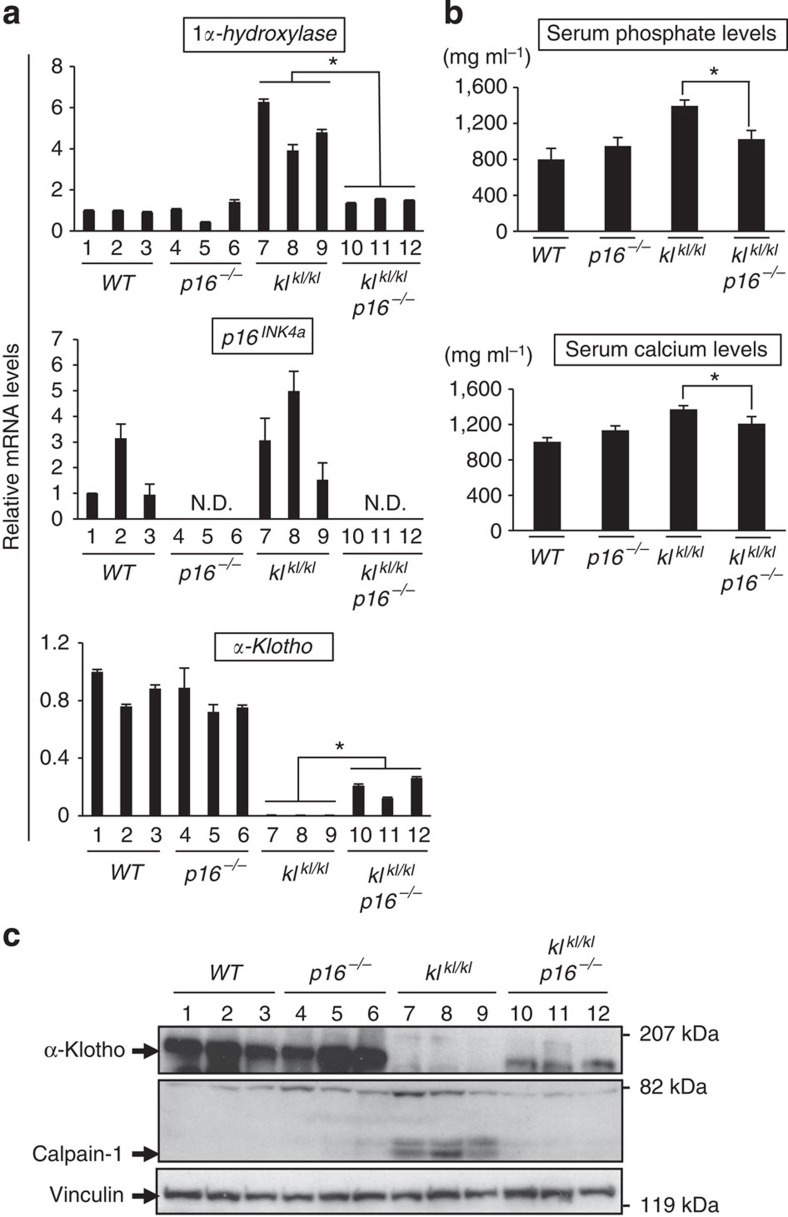
Recovery of *α-klotho* expression in the kidney by *p16*^*INK4a*^ ablation. (**a**) The relative levels of indicated mRNA in kidneys of 2- to 6-month-old male mice of each genotype were examined using RT–qPCR. Representative results of three individual male mice were shown. N.D. represents ‘not detected'. (**b**) Serum phosphate and serum calcium levels of 2- to 6-month-old male mice of each genotype were shown. Representative results of three individual mice were shown. (**c**) Kidneys of each genotype were subjected to western blot analysis using antibodies shown left. Calpain-1 represents the levels of activated form of Calpain-1. Vinculin was used as a loading control. Representative results of three individual male mice were shown. For all graphs, the experiments were performed in triplicate, and representative results from three independent experiments are shown. Data were analysed by Welch's *t*-test and are displayed as mean±s.d. *P<0.05.

**Figure 5:**
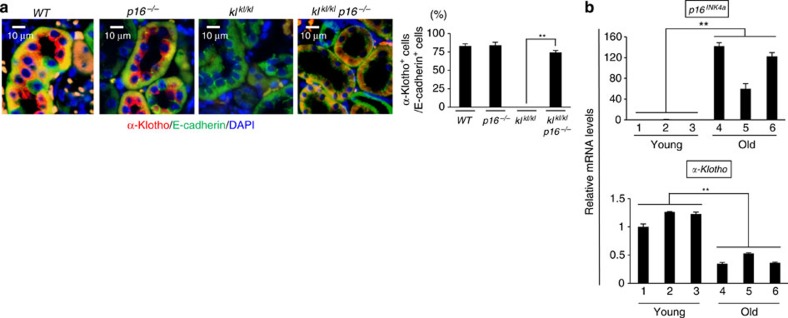
Inverse correlation between *α-klotho* and *p16*^*INK4a*^ expression. (**a**) Immunohistochemical analysis of *α*-klotho (red), E-cadherin (green) and 4,6-diamidino-2-phenylindole (blue) in 11-week-old male mouse kidney sections of each genotype. Representative results of three individual male mice were shown. The histograms indicate the percentages of E-cadherin-expressing cells that were positive for *α*-klotho expression. At least 100 cells were scored per group. (**b**) The relative levels of indicated mRNA in kidneys of young (10- to 20-week-old) or old (120- to 140-week-old) *Wt* male mice were examined using RT–qPCR. Representative results of three individual male mice were shown. For all graphs, the experiments were performed in triplicate, and representative results from three independent experiments are shown. Data were analysed by Welch's *t*-test and are displayed as mean±s.d. *P<0.05, **P<0.01.

To explain the effects of the *p16*^*INK4a*^ ablation on the accelerated-ageing phenotypes of *kl*^*kl/kl*^ mice, we next took a closer look at the biochemical characteristics of *kl*^*kl/kl*^ mice. In *kl*^*kl/kl*^ mice, the level of *1α-hydroxylase* gene expression is reportedly increased in the kidneys, resulting in an elevated serum level of 1,25-dihydroxyvitamin D, the active metabolite of vitamin D that regulates calcium and phosphate homeostasis^30^. Since these changes are known to be associated with the accelerated-ageing phenotypes in *kl*^*kl/kl*^ mice^31^, we examined the levels of these biochemical hallmarks in *kl*^*kl/kl*^ mice. Notably, the levels of these hallmarks were substantially reduced in *kl*^*kl/kl*^ *p16*^*−/−*^ mice, as compared with those in *kl*^*kl/kl*^ mice (Fig. 4a,b). Moreover, the aberrant activation of calpain-1 and the ectopic calcification in kidneys, signs of the abnormal calcium homeostasis observed in *kl*^*kl/kl*^ mice^20^,^27^,^32^, were absent in *kl*^*kl/kl*^ *p16*^*−/−*^ mice (Figs 2 and 4c), implying that the α-klotho function might be somewhat restored in the *kl*^*kl/kl*^ *p16*^*−/−*^ mice. Since the *kl*^*kl/kl*^ mice are not a complete null, but have a severe hypomorphic mutation for *α-klotho* expression, the entire *α-klotho*-coding sequence is intact in *kl*^*kl/kl*^ mice^20^. Thus, we next wondered whether *p16*^*INK4a*^ ablation could restore the levels of *α-klotho* expression in *kl*^*kl/kl*^ mice. Indeed, the levels of both *α-klotho* mRNA and protein were substantially increased in the kidneys of *kl*^*kl/kl*^ *p16*^*−/−*^ mice, as compared with those in *kl*^*kl/kl*^ mice, albeit to lesser extents as compared with those in wt mice (Fig. 4a,c). Notably, *α-klotho* expression was observed only in the renal distal convoluted tubules in *kl*^*kl/kl*^ *p16*^*−/−*^ mice (Fig. 5a), which are the major sources of *α-klotho* expression in wt mice^20^. Thus, it appears that *p16*^*INK4a*^ ablation restores the normal *α-klotho* expression pattern in *kl*^*kl/kl*^ mice. Importantly, in stark contrast to the *kl*^*kl/kl*^ mice, the *p16*^*INK4a*^ ablation failed to reverse the accelerated-ageing phenotype in mice lacking the *α*-*klotho* gene (*α*-*klotho* knockout mice (*kl*^*−/−*^; ref. 30; Supplementary Fig. 3). These results indicate that *p16*^*INK4a*^ ablation mitigates the accelerated-ageing phenotypes of *kl*^*kl/kl*^ mice, by restoring *α*-*klotho* expression.

### p16^INK4a^ downregulates *α-klotho* expression in wt mice

The obvious next question is whether p16^INK4a^ downregulates *α-klotho* expression in wt mice. Note that there is an inverse correlation between the levels of *p16*^*INK4a*^ expression and *α-klotho* expression during the ageing process in kidneys (Fig. 5b). However, because *p16*^*−/−*^ mice die prematurely from cancer^17^ (see also Fig. 1b), we cannot examine whether *p16*^*INK4a*^ ablation ameliorates the ageing-associated decline of *α-klotho* expression in mice harbouring wt *α-klotho*. To circumvent this problem, we employed the mouse model of chemically induced kidney injury. It was previously reported that patients with chronic renal failure develop multiple age-related disorders resembling those of *kl*^*kl/kl*^ mice, with a marked reduction of *α-klotho* expression in kidneys^33^,^34^. Moreover, treatment with cisplatin, a chemotherapeutic agent that causes severe adverse actions with nephrotoxicity, is known to provoke a significant reduction of *α*-*klotho* expression in kidneys, accompanied by the accumulation of DNA damage^35^. Since persistent DNA damage induces *p16*^*INK4a*^ expression in many different cell types^12^, we analysed the effect of p16^INK4a^ expression on the levels of *α*-*klotho* expression in the cisplatin-induced kidney injury model. Indeed, the cisplatin treatment resulted in a marked reduction of *α*-*klotho* expression in the renal distal convoluted tubules of wt mice, coinciding with the accumulation of γH2AX foci, a sign of the DNA damage response, and the induction of *p16*^*INK4a*^ expression (Supplementary Fig. 4). Notably, however, the cisplatin-induced reduction of *α*-*klotho* expression was substantially attenuated in the *p16*^*−/−*^ mice (Supplementary Fig. 4), although this level of *α*-*klotho* restoration was insufficient to block the cisplatin-induced nephrotoxicity in this experimental condition (Supplementary Fig. 5), indicating that p16^INK4a^ has the potential to downregulate *α-klotho* expression in wt mice as well.

### p16^INK4a^ downregulates the *α*-*klotho* promoter in murine cells

**Figure 6:**
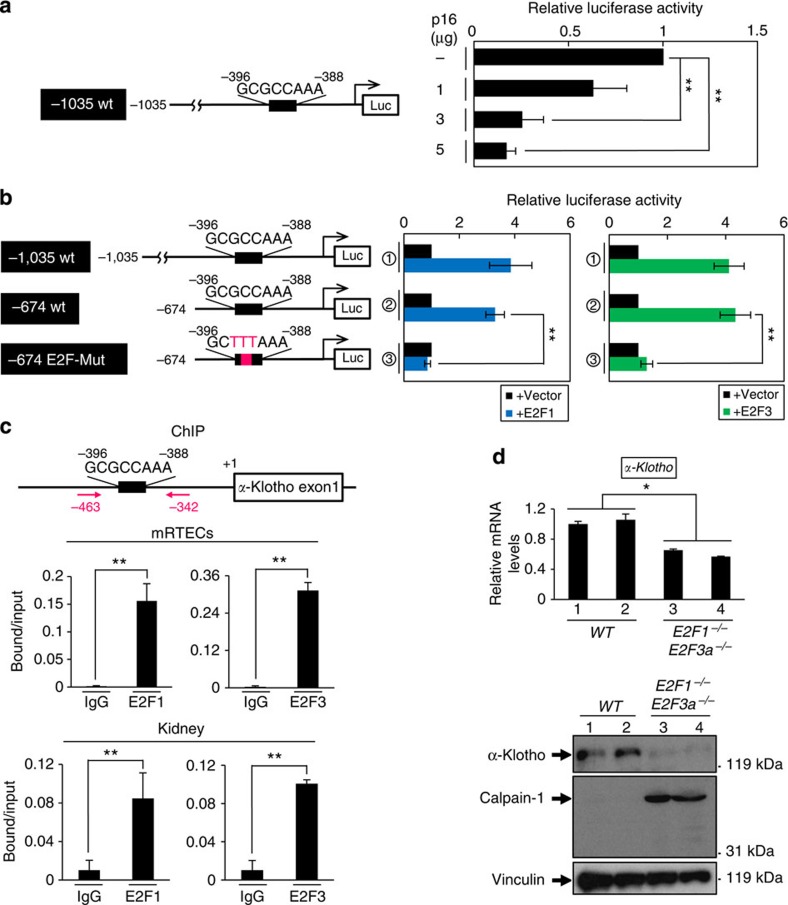
p16^INK4a^ downregulates *α-klotho* gene promoter activity *via* E2F. (**a**,**b**) Schematic representation of the reporter construct of mouse *α-klotho* gene promoter used in the analysis (left panel). The E2F-binding element is shown as a black rectangle with the sequence and firefly luciferase is shown as Luc. The reporter construct was co-transfected into mRTECs along with LacZ or Renilla plasmid. Where indicated, cells were also co-transfected with an increasing amount of p16^INK4a^ expression plasmid (**a**) or with 2 μg of expression plasmid encoding E2F1 or E2F3 (**b**). (**c**) Early-passage mRTECs and kidney tissues were prepared from 6- to 10-week-old male mice and were subjected to ChIP analysis using antibodies shown at bottom and PCR primers shown at the top (red arrows). (**d**) The relative levels of *α-klotho* mRNA and protein expression in kidneys of each genotype were examined using RT–qPCR (upper panel) or western blot analysis (lower panel). Representative results of two individual 2.7-week-old male mice were shown. For all graphs, the experiments were performed in triplicate, and representative results from three independent experiments are shown. Data were analysed by Welch's *t*-test and are displayed as mean±s.d. *P<0.05, **P<0.01.

**Figure 7:**
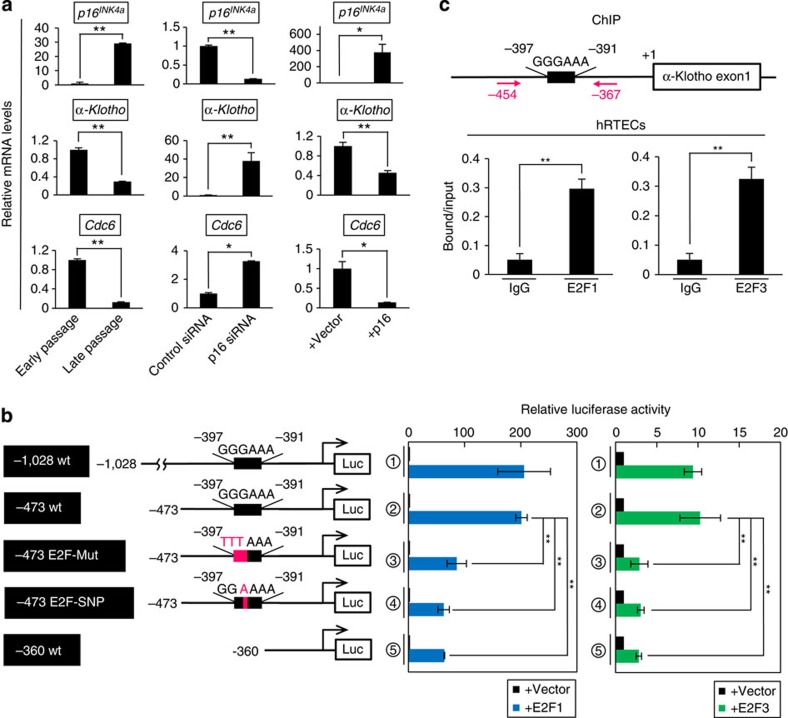
p16^INK4a^ downregulates *α-klotho* expression in humans. (**a**) The relative levels of indicated mRNA in hRTECs were examined using RT–qPCR. RNAs were prepared from early-passaged or late-passaged hRTECs (left panels), early-passaged hRTECs transfected with siRNA against p16^INK4a^ or control (middle panels), or early-passaged hRTECs transfected with or without p16^INK4a^ expression vector (right panels). (**b**) Schematic representation of the reporter construct of the human *α-klotho* gene promoter used in the analysis (left panel). The E2F-binding element is shown as a black rectangle with the sequence and firefly luciferase is shown as Luc. The reporter construct was co-transfected into hRTECs along with Renilla plasmid. Where indicated, cells were also co-transfected with 1.5 μg of expression plasmid encoding E2F1 or E2F3. (**c**) Early-passage cultured hRTECs were subjected to ChIP analysis using antibodies shown at bottom and PCR primers shown at the top (red arrows). For all graphs, the experiments were performed in triplicate, and representative results from three independent experiments are shown. Data were analysed by Welch's *t*-test and are displayed as mean±s.d. *P<0.05, **P<0.01.

To further verify this notion, we next sought evidence that p16^INK4a^ downregulates *α*-*klotho* promoter activity. As *α-klotho* expression is rather limited in the renal distal convoluted tubules^20^,^24^, we were unable to find any established murine cell lines expressing substantial levels of *α-klotho* (Supplementary Fig. 6). Therefore, primary mouse renal tubular epithelial cells (mRTECs) were prepared from the kidneys of wt mice, and were used for a promoter–reporter analysis. The luciferase activity of a reporter plasmid containing the region 1,035 nucleotides upstream of the mouse *α-klotho* translation start site was reduced by the ectopic expression of *p16*^*INK4a*^ in a dose-dependent manner in early-passage primary mRTECs (Fig. 6a), indicating that p16^INK4a^ indeed downregulates *α-klotho* expression at the promoter level. Although the *α-klotho* promoter sequences are not well conserved between human and mouse, both include potential binding sites for E2F transcription factors, which are critical downstream mediators of the p16^INK4a^–retinoblastoma tumour suppressor pathway^6^, at the same position from the *α-klotho* translation start sites (Figs 6a and 7b). Notably, newborn mice lacking both E2F1 and E2F3a, a subset of the activator E2Fs (referred to as *E2F1*^*−/−*^ *E2F3a*^*−/−*^ mice), reportedly exhibited normal weight and appearance; however, by their third week of life the proliferative index of most tissues was significantly reduced, and 90% of the mice became severely runted and died within 2 months^36^. Furthermore, white adipose tissues were absent and lung alveolar branching was severely reduced in *E2F1*^*−/−*^ *E2F3a*^*−/−*^ mice^36^. Since these phenotypes are reminiscent of the *kl*^*kl/kl*^ phenotypes^20^, we analysed whether E2F1 and/or E2F3 activate the *α-klotho* promoter activity. Indeed, the ectopic expression of either E2F1 or E2F3 increased the activity of the *α-klotho* promoter in cultured primary mRTECs (Fig. 6b). However, this was not the case when the E2F-binding element was disrupted by a nucleotide substitution in the reporter plasmid (Fig. 6b, -674 E2F-Mut). Moreover, increasing amounts of E2F3 blocked the *trans*-repressing activity of co-transfected p16^INK4a^ in early-passage primary mRTECs (Supplementary Fig. 7). Note that endogenous E2F1 and E2F3 were found to bind to the *α-klotho* promoter, as judged by a chromatin immunoprecipitation (ChIP) analysis using cultured primary mRTECs or kidney tissues prepared from wt mice (Fig. 6c). These results, in conjunction with the observation that the levels of endogenous *α-klotho* mRNA and protein expression were substantially reduced in the kidneys of *E2F1*^*−/−*^ *E2F3a*^*−/−*^ mice (Fig. 6d), strongly suggest that p16^INK4a^ downregulates *α-klotho* expression, at least partly by blocking the function of activator E2Fs in wt mice.

### p16^INK4a^ downregulates *α*-*klotho* expression in human cells

Finally, to further support our murine data and to extend the analysis to human physiology, we tested whether p16^INK4a^ downregulates *α*-*klotho* mRNA expression in human cells. Similar to murine cells, primary human RTECs (hRTECs), but not other human cell lines, express substantial levels of *α*-*klotho* (Supplementary Fig. 8). We thus used primary hRTECs in the following experiments. We found that the levels of *α*-*klotho* mRNA expression declined when cultured primary hRTECs were rendered senescent by serial passage, accompanied by the induction of *p16*^*INK4a*^ mRNA expression (Fig. 7a, left). However, the short interfering RNA (siRNA)-mediated depletion of *p16*^*INK4a*^ substantially increased the levels of *α*-*klotho* mRNA expression in late-passage hRTECs, coinciding with the increased expression of *cdc6,* an established E2F target gene (Fig. 7a, middle). Conversely, the ectopic expression of *p16*^*INK4a*^ reduced the levels of *α*-*klotho* mRNA expression in early-passage hRTECs (Fig. 7a, right). Together, these results suggest that *p16*^*INK4a*^ downregulates *α-klotho* expression, by blocking the function of the activator E2Fs in human cells, as well as in mouse cells. Indeed, the ectopic expression of either E2F1 or E2F3 substantially increased the transcriptional activity of the human *α-klotho* promoter in cultured hRTECs (Fig. 7b, −1,028 wt and −473 wt). These effects were blunted when the putative E2F-binding site within the human *α-klotho* promoter was mutated or deleted in the reporter plasmid (Fig. 7b, -473 E2F-Mut and-360 wt). Furthermore, the ChIP analysis revealed that endogenous E2F1 and E2F3 bind to the human *α-klotho* promoter in cultured hRTECs (Fig. 7c). Interestingly, the G to A single-nucleotide polymorphism (SNP), in the putative E2F-binding site of the human *α-klotho* promoter, reportedly impaired the DNA–protein interaction and is associated with the reduction of bone mineral density (BMD) in aged postmenopausal women^37^. Indeed, the G to A substitution in the E2F-binding site of the reporter plasmid greatly reduced the response to E2F overexpression (Fig. 7b,-473 E2F-SNP). These results, in conjunction with previous observations that there is the potential inverse correlation between the levels of renal *p16*^*INK4a*^ expression and *α*-klotho expression in elderly people^21^,^38^,^39^,^40^, suggest that *p16*^*INK4a*^ is likely to have the potential to downregulate *α-klotho* expression by blocking the transcriptional activity of E2Fs in human kidneys.

## Discussion

**Figure 8:**
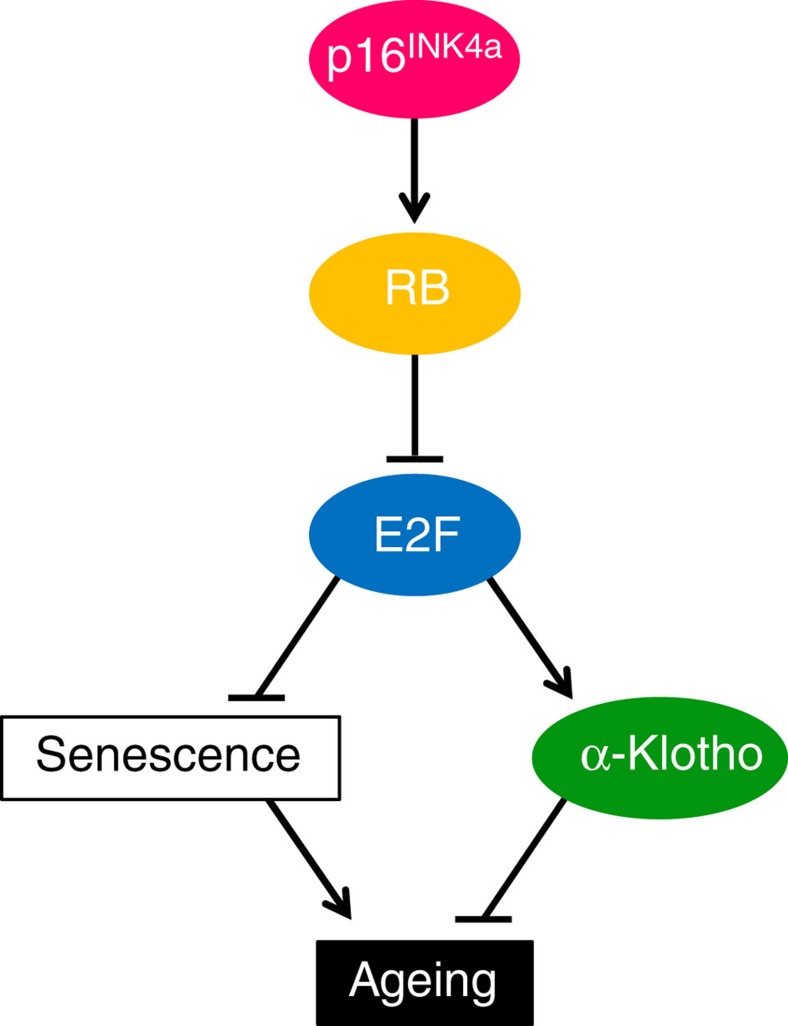
Dual roles for the p16^INK4a^ RB pathway in organismal ageing. The p16^INK4a^ has an established role in provoking cellular senescence, which is likely to cause stem cell ageing and thereby contributing to organismal ageing. Here we show that, in addition, p16^INK4a^ also contributes to organismal ageing through blocking the expression of ageing suppressor, *α*-klotho.

The ageing process is multifactorial, with genetic background and environmental stress as two critical components^4^,^41^. The mutation of the *α-klotho* gene causes multiple premature ageing phenotypes, including a shortened lifespan in mice^20^, and some SNPs in the human *α-klotho* gene are associated with reduced lifespans^37^,^42^,^43^. Moreover, the levels of plasma *α*-klotho decrease with increasing age and are associated with longevity in humans^21^,^40^, indicating that the *α-klotho* gene is an important antiageing gene in both mouse and human. However, it remained unclear how the *α-klotho* gene could be linked to environmental stress. Here we show that the *p16*^*INK4a*^ tumour-suppressor gene, a stress sensor known to induce cellular senescence^5^,^6^,^7^,^8^,^9^,^10^,^11^,^12^, downregulates *α-klotho* gene expression in both mouse and human renal tubular epithelial cells. Ablation of the *p16*^*INK4a*^ gene mitigates various accelerated-ageing phenotypes of *kl*^*kl/kl*^ mice, including shortened maximum lifespan, by partially restoring *α*-*klotho* expression (Figs 1, 2 and 4). Furthermore, cell culture studies reveal that p16^INK4a^ represses *α*-*klotho* gene expression at the promoter level by blocking the function of activator E2F, most likely through activation of retinoblastoma protein in mouse and human cells (Figs 6, 7, 8). These results, together with previous epidemiological studies^38^,^39^, suggest that this previously unrecognized function of p16^INK4a^ is likely to play a role in humans as well as in mice.

However, p16^INK4a^ has an established role in the implementation of cellular senescence in stem/progenitor cells^5^,^6^,^7^,^8^,^9^,^10^, thereby causing dysfunctional tissue regeneration and repair^13^, which are likely to reduce longevity. Indeed, a series of studies using middle-aged *p16*^*−/−*^ mice revealed that the ageing-associated induction of *p16*^*INK4a*^ expression reduces the proliferative and regenerative capacities of certain progenitor cells during the ageing process^14^,^15^,^16^, further illustrating the importance of the p16^INK4a^-cellular senescence pathway in the development of ageing phenotypes^44^. Nevertheless, our present study revealed that p16^INK4a^ plays another role in promoting ageing phenotypes, through the downregulation of the expression of the *α*-klotho ageing suppressor (see model in Fig. 8). This previously unrecognized pathway, linking *p16*^*INK4a*^ to *α-klotho* expression, enhances our understanding of the molecular mechanisms underlying the development and progression of ageing phenotypes in mammals and opens up new possibilities for their control.

## Methods

### Mice

All efforts were made to minimize animal suffering and to reduce the number of animals used. Wt mice (C57BL/6J), *klotho* (*kl*^*kl/kl*^) mice^20^ (mixed C57BL/6J and C3H/J genetic background; 50% C57BL/6J, 50% C3H/J) and *α*-*klotho* knockout (*kl*^*−/−*^) mice (C57BL/6J) were purchased from CLEA Inc., Japan. *Kl*^*kl/kl*^ mice were backcrossed with C57BL/6J mice for five generations and used in this study. *Kl*^*kl/kl*^ *p16-luc* mice were generated by crossing *kl*^*kl/kl*^ mice with *p16-luc* mice^12^. *E2F1*^*−/−*^ *E2F3*^*−/−*^ mice^36^ were provided by Dr Gastabo Leone. *p16*^*−/−*^ mice^45^ were provided by Dr Norman E. Sharpless. These mice were maintained under specific pathogen-free conditions, on a 12-h light–dark cycle and fed normal diet (CE-2 from CLEA Inc., composed of 12 kcal% fat, 29 kcal% protein and 59 kcal% carbohydrates). All animal experiments were cared for by protocols approved by the Committee for the Use and Care of Experimental Animals of the Japanese Foundation for Cancer Research.

### Bone radiography

BMD was analysed using X-ray radiography. Femur and tibia were resected from 11-week female mice, and placed on wrapped films (FUJIFILM INDUSTRIAL X-RAY FILM ENVELOPAK IX FR; FUJIFILM Corporation, Japan), and exposed to X-irradiation at 20 kVp, 9 mA for 15 s using a SOFTEX CMB-2 (SOFTEX CO., LTD., Japan). Films were developed using a Fuji Medical Films Processor SEPROS SV (FUJIFILM Corporation) and inspected for BMD.

### Bioluminescence imaging

For the detection of luciferase expression, mice were anaesthetized, injected intraperitoneally with D-luciferin sodium salt (75 mg kg^−g^) 5 min before beginning photon recording. Mice were placed in the light-tight chamber and a grey-scale image of the mice was first recorded with dimmed light followed by acquisition of luminescence image using a cooled CCD (charged-coupled device) camera (PIXIS 1024B; Princeton Instruments)^12^,^46^. The signal-to-noise ratio was increased by 2 × 2 binning and 5-min exposure. For colocalization of the luminescent photon emission on the animal body, grey scale and pseudo-colour images were merged by using IMAGE-PRO PLUS (Media Cybernetics).

### Histology and immunofluorescence analysis

Samples were fixed in 10% formalin for a 24 h or longer, progressively dehydrated through gradients of alcohol and embedded in paraffin. Samples were then sectioned on a microtome (5-μm thick), deparaffinized in xylene, rehydrated and then stained with haematoxylin and eosin (HE). For immunofluorescence, the relevant Alexa Fluor 488 goat anti-mouse or 546 goat anti-rabbit antibodies (1:1,000, Molecular probes) were used for detection of primary antibodies. Fluorescence images were observed and photographed using an immunofluorescence microscope (Carl Zeiss). The primary antibodies used for mouse samples were as follows: Klotho (1:100, TransGenic Inc., KO603), E-cadherin (1:100, Cell Signaling no. 3195), γ-H2AX (1:100, Millipore, 05-636). E-cadherin was used as a marker of the renal distal tubes^47^,^48^,^49^. Calcium deposition was visualized with the von Kossa staining. Paraffin-embedded sections were deparaffinized and rehydrated. Fixed sections were incubated with 5% silver nitrate during exposure to light for 60 min, and washed with distilled water. Excess silver was washed out with 5% sodium thiosulfate for 2 min. The sections were then stained with Kernechtrot dye. Epidermal and subcutaneous fat-layer thickness and intestinal villi length were determined using 10–74 random measurements along the length of skin and small intestine from at least three mice per age group and genotype. For skin sections, the skin was cut parallel to the spine and sections were cut perpendicular to the skin surface. For villi sections, intestinal tracts were flushed with PBS and rolled up in a compact circle using longitudinally oriented jejunal sections for analysis; 5-μM sections were used for HE staining, and the Image J software was used for length measurements^50^. The mean linear intercept (Lm) in the lung tissue was calculated using light microscopy and Image J software. An overlay consisting of horizontal and vertical, parallel lines was placed over the photographed image of each region. All intercepts with alveolar septal tissue were counted. The total length of all the lines together divided by the number of intercepts gives the Lm for the region studied. The overall mean of the Lm for each of the three regions studied for each tissue block was used as the Lm for the corresponding tissue block^51^.

### Reverse transcription and quantitative real-time PCR

Total RNA was extracted from mouse tissues using TRIzol reagent (Life Technologies). Reverse transcription and quantitative real-time PCR (RT–qPCR) was performed using the SYBER Premix EX Taq system (TAKARA) and a Prism 7900HT (ABI)^52^,^53^. Amplified signals were confirmed to be single bands with gel electrophoresis and were normalized to the levels of glyceraldehyde 3-phosphate dehydrogenase. The data were analysed using the SDS2.1 software (ABI)^46^. The PCR primer sequences used are shown in the Supplementary Table 1.

### Measurement of serum phosphate, calcium and BUN

The concentration of serum phosphate, calcium and blood urea nitrogen (BUN) was measured with a phosphate assay kit (Serotec UPi-L; Serotec Co. Ltd., Japan), a calcium assay kit (Metalloassay Ca-CPZIII; AKJ Global Technology, Japan) and a BUN assay kit (Iatoro LQ UN rate (A) II; LSI medience Co. Ltd., Japan), respectively, according to the manufacturers' instruction.

### Cell culture

For primary mRTECs^47^, kidneys of 6- to 10-week-old male mice were taken and placed in cold PBS containing antibiotics (Penicillin and streptomycin). The medulla of the kidneys were removed and the cortex of the kidney was taken, minced and transferred to 2 ml serum-free DMEM containing 0.1% collagenase (Sigma). Minced kidney cortex tissue was incubated at 37 °C with shaking for 30 min. The tissue suspension was mixed with 10 ml of DMEM containing 10% serum to inactivate collagenase. The remaining tissue mass was removed through the 70-μm strainer, and cells were pelleted using centrifugation. The cell number was counted and seeded on the poly-L-lysin-coated dishes (Corning, USA) for ChIP analysis or for transfection. For normal hRTECs, primary hRTECs were purchased from Lonza (Lonza, Switzerland) and were cultured according to the manufacturer's instruction.

### Western blot analysis

Tissue lysates were prepared using a homogenizer in lysis buffer (50 mM HEPES (pH 7.5), 150 mM NaCl, 1 mM EDTA, 2.5 mM EGTA, 10% glycerol, 0.1% Tween 20 and 10 mM β-glycerophosphate) containing protease inhibitor cocktail (Nacalai tesque)^54^. The transferred membranes were immunoblotted directly with following antibodies, and the signals were detected using enhanced chemiluminescence system (GE Healthcare). The first antibodies used were as follows: *α*-Klotho (1:500, TransGenic Inc., KO603), Calpain-1 (μ-Calpain; 1:1,000, Enzo ALX-804-050-R100), α-tubulin (1:1,000, Sigma T9026) and Vinculin (1:1,000, Sigma V9131). Expanded immunoblots are shown in Supplementary Figs 9–11.

### ChIP analysis

ChIP analysis was performed using the EZ-ChIP kit (Millipore) according to the manufacturer's instruction. The immunoprecipitation of cross-linked chromatin was conducted with anti-mouse E2F1 (1:1,000, Santa Cruz, sc-193X), anti-mouse E2F3 (1:1,000, Santa Cruz, sc-878X), anti-human E2F1 (1:1,000, Santa Cruz, sc-193X), anti-human E2F3 (1:1,000, Santa Cruz, sc-878X) and rabbit IgG (1:1,000, Cell Signaling Technology, 2729) as a negative control. After immunoprecipitation, DNA was extracted using the QIAquick PCR purification kit (Qiagen) and an aliquot was amplified using qPCR using following primers flanking the putative mouse E2F-binding site position at −388 to −396 bp of mouse *α-klotho gene* promoter: 5′-TGTTCTCTGAAAGATTCCCC-3′ and 5′-TCCCTTTGCCTTCCTGGGAC-3′, or primers flanking the putative human E2F-binding site position at −391 to −397 bp of human *α-klotho gene* promoter: 5′-TGGGAGAAAAGTGAGAGCAG-3′ and 5′-TGGGAGAAAAGTGAGAGCAG-3′.

### *In vivo* ChIP analysis

Sixty milligrams of kidney tissue was chopped into 1- to 2-mm pieces using razor blades and were transferred into a new tube containing 1 ml PBS with proteinase inhibitor cocktail (Nacalai tesque). Crosslinking was performed in 1% of formaldehyde by rotating at room temperature for 15 min. This crosslinking reaction was stopped by adding fresh glycine to a final concentration of 0.125 M by continuous rotation at room temperature for 5 min. Tissue pieces were then washed and suspended in PBS-containing proteinase inhibitor cocktail, and were grinded by Dounce homogenizing seven times. The cell pellet was resuspended into Lysis buffer containing 5 mM PIPES pH8.0, 85 mM KCl, 0.5% NP40 and proteinase inhibitor cocktail, and lysed by Dounce homogenizing four times to aid in nuclei release^52^. After this procedure, ChIP was performed using the EZ-ChIP kit (Millipore). The immunoprecipitation of cross-linked chromatin was conducted with anti-mouse E2F1 (1:1,000, Santa Cruz, sc-193X), anti-mouse E2F3 (1:1,000, Santa Cruz, sc-878X) and rabbit IgG (1:1,000, Cell Signaling Technology, 2729) as a negative control. After immunoprecipitation, DNA was extracted using the QIAquick PCR purification kit (Qiagen), and an aliquot was amplified by qPCR using the following primers flanking the putative mouse E2F-binding site position at −388 to −396 bp of mouse *α*-*klotho gene* promoter: 5′-TGTTCTCTGAAAGATTCCCC-3′ and 5′-TCCCTTTGCCTTCCTGGGAC-3′.

### RNA interference

RNA interference (RNAi) was performed using the RNAiMAX transfection reagent (Life Technologies) and siRNA oligos against target genes^53^. The sequences of targeting oligo are as follows.

Human p16 5′-GAGGAGGUGCGGGCGCUGCTT-3′ (sense) and 5′-GCAGCGCCCGCACCUCCUCTT-3′ (antisense) Control 5′-AUGAACGUGAAUUGCUCAATT-3′ (sense) and 5′-UUGAGCAAUUCACGUUCAUTT-3′ (antisense).

### Luciferase–reporter assays

The human and mouse *α-klotho* gene promoter sequence was amplified with PCR using genomic DNA extracted from the mouse tail or BAC clone (RP11-720E2) containing the entire human *α-klotho* gene as templates. Deletion mutants were prepared with standard PCR procedures. Promoter sequences containing point mutations were generated using the Quick Change Site-directed Mutagenesis kit (Agilent Technologies). The promoter fragments were inserted into PGL3 basic firefly luciferase reporter plasmid (Promega). All inserted DNAs were sequenced and verified. Transfection of reporter plasmids was performed using the X-treamGENE9 DNA transfection reagent (Roche) according to the manufacturer's instructions. The luciferase assays were performed using the Luciferase assay systems kit (Promega). Cytomegalovirus promoter-renilla luciferase plasmid or SV40 promoter-β-galactosidase plasmid was used as an internal control.

### Cisplatin (*cis*-diamminedichloro-platinum II) treatment

Cisplatin was purchased from Wako pure chemical, Japan and dissolved in saline. Mice were intraperitoneally injected with Cisplatin solution (12 mg kg^−g^) three times (every other day) for a week and killed. The kidneys were immediately taken from mice and used for analysis.

### Statistical analysis

The significance of differences was analysed by Student's *t*-test, Welch's *t*-test or Mann–Whitney *U*-test. *P* values of less than 0.05 were considered to be significant. Statistical analyses were performed using the GraphPad Prism programme Ver. 5.01 (*GraphPad* Software Inc.).

## Author contributions

N.O. and E.H. designed the experiments. S.S., Y.K., A.T., Y.I., A.H., A.O., M.T., K.Y. and N.O. performed experiments. H.S. helped in calcium analysis. H.K. and Y.I. helped in histopathology. S.S., Y.N., N.O. and E.H. analysed the data. E.H. wrote the manuscript.

## Additional information

**How to cite this article:** Sato, S. *et al.* Ablation of the p16^INK4a^ tumour suppressor reverses ageing phenotypes of *klotho* mice. *Nat. Commun.* 6:7035 doi: 10.1038/ncomms8035 (2015).

## Supplementary Material

Supplementary Information Supplementary Figures 1-11 and Supplementary Table 1

## References

[b1] Cheung T. H. & Rando T. A. Molecular regulation of stem cell quiescence. Nat. Rev. Mol. Cell Biol. 14, 329–340 (2013). 23698583 10.1038/nrm3591 PMC3808888

[b2] Sharpless N. E. & DePinho R. A. How stem cells age and why this makes us grow old. Nat. Rev. Mol. Cell Biol. 8, 703–713 (2007). 17717515 10.1038/nrm2241

[b3] Sperka T., Wang J. & Rudolph K. L. DNA damage checkpoints in stem cells, ageing and cancer. Nat. Rev. Mol. Cell Biol. 13, 579–590 (2012). 22914294 10.1038/nrm3420

[b4] López-Otín C., Blasco M. A., Partridge L., Serrano M. & Kroemer G. The hallmarks of aging. Cell 153, 1194–1217 (2013). 23746838 10.1016/j.cell.2013.05.039 PMC3836174

[b5] Kim W. Y. & Sharpless N. E. The regulation of *INK4/ARF* in cancer and aging. Cell 127, 265–275 (2006). 17055429 10.1016/j.cell.2006.10.003

[b6] Gil J. & Peters G. Regulation of the *INK4b-ARF-INK4a* tumour suppressor locus: all for one or one for all. Nat. Rev. Mol. Cell Biol. 7, 667–677 (2006). 16921403 10.1038/nrm1987

[b7] Collado M., Blasco M. A. & Serrano M. Cellular senescence in cancer and aging. Cell 130, 223–233 (2007). 17662938 10.1016/j.cell.2007.07.003

[b8] Campisi J. & d'Adda di Fagagna F. Cellular senescence: when bad things happen to good cells. Nat. Rev. Mol. Cell Biol. 8, 729–740 (2007). 17667954 10.1038/nrm2233

[b9] Jessie C. & Sedivy J. M. Cellular senescence and organismal aging. Mech. Ageing Dev. 129, 467–474 (2008). 18502472 10.1016/j.mad.2008.04.001 PMC3297662

[b10] Kuilman T., Michaloglou C., Mooi W. J. & Peeper D. S. The essence of senescence. Genes Dev. 24, 2463–2479 (2010). 21078816 10.1101/gad.1971610 PMC2975923

[b11] Krishnamurthy J. *et al.* *Ink4a/Arf* expression is a biomarker of aging. J. Clin. Invest. 114, 1299–1307 (2004). 15520862 10.1172/JCI22475 PMC524230

[b12] Yamakoshi K. *et al.* Real-time *in vivo* imaging of p16^Ink4a^ reveals cross talk with p53. J. Cell Biol. 186, 393–407 (2009). 19667129 10.1083/jcb.200904105 PMC2728398

[b13] Sousa-Victor P. *et al.* Gereiatric muscle stem cells switch reversible quiescence into senescence. Nature 506, 316–321 (2014). 24522534 10.1038/nature13013

[b14] Janzen V. *et al.* Stem-cell ageing modified by the cyclin-dependent kinase inhibitor p16^INK4a^. Nature 443, 421–426 (2006). 16957735 10.1038/nature05159

[b15] Krishnamurthy J. *et al.* p16^INK4a^ induces an age-dependent decline in islet regenerative potential. Nature 443, 453–457 (2006). 16957737 10.1038/nature05092

[b16] Molofsky A. *et al.* Increasing p16^INK4a^ expression decreases forebrain progenitors and neurogenesis during ageing. Nature 443, 448–452 (2006). 16957738 10.1038/nature05091 PMC2586960

[b17] Sharpless N. E., Ramsey M. R., Balasubramanian P., Castrillon D. H. & DePinho R. A. The differential impact of p16^INK4a^ or p19^ARF^ deficiency on cell growth and tumorigenesis. Oncogene 23, 379–385 (2004). 14724566 10.1038/sj.onc.1207074

[b18] Molofsky A. V., He S., Bydon M., Morrison S. J. & Pardal R. Bmi-1 promotes neural stem cell self-renewal and neural development but not mouse growth and survival by repressing the p16^Ink4a^ and p19^Arf^ senescence pathway. Genes Dev. 19, 1432–1473 (2005). 15964994 10.1101/gad.1299505 PMC1151659

[b19] Baker D. J. *et al.* Opposing roles for p16^Ink4a^ and p19^Arf^ in senescence and ageing caused by BubR1 insufficiency. Nat. Cell Biol. 10, 825–836 (2008). 18516091 10.1038/ncb1744 PMC2594014

[b20] Kuro-o M. *et al.* Mutation of the mouse *klotho* gene leads to a syndrome resembling ageing. Nature 390, 45–51 (1997). 9363890 10.1038/36285

[b21] Yamazaki Y. *et al.* Establishment of sandwitch ELISA for soluble alpha-Klotho measurement: age-dependent change of soluble alpha-Klotho levels in healthy subjects. Biochem. Biophys. Res. Commun. 398, 513–518 (2010). 20599764 10.1016/j.bbrc.2010.06.110 PMC4130489

[b22] Kurosu H. *et al.* Suppression of aging in mice by the hormone Klotho. Science 309, 1829–1833 (2005). 16123266 10.1126/science.1112766 PMC2536606

[b23] Kuro-o M. Klotho and aging. Biochim. Biophys. Acta 1790, 1049–1058 (2009). 19230844 10.1016/j.bbagen.2009.02.005 PMC2743784

[b24] Chang Q. *et al.* The β-Glucuronidase Klotho hydrolyzes and activates the TRPV5 channel. Science 310, 490–493 (2005). 16239475 10.1126/science.1114245

[b25] Shiraki-Iida T. *et al.* Structure of the mouse klotho gene and its two transcripts encoding membrane and secreted protein. FEBS Lett. 424, 6–10 (1998). 9537505 10.1016/s0014-5793(98)00127-6

[b26] Urakawa I. *et al.* Klotho converts canonical FGF receptor into a specific receptor for FGF23. Nature 447, 770–774 (2006). 17086194 10.1038/nature05315

[b27] Imura A. *et al.* α-Klotho as a regulator of calcium homeostasis. Science 316, 1615–1618 (2007). 17569864 10.1126/science.1135901

[b28] Liu H. *et al.* Augmented Wnt signaling in a mammalian model of accelerated aging. Science 317, 803–806 (2007). 17690294 10.1126/science.1143578

[b29] Eren M. *et al.* PAI-1-regulated extracellular proteolysis governs senescence and survival in *klotho* mice. Proc. Natl Acad. Sci. USA 111, 7090–7095 (2014). 24778222 10.1073/pnas.1321942111 PMC4024885

[b30] Tsujikawa H., Kurotaki Y., Fujimori T., Fukuda K. & Nabeshima Y. *Klotho*, a gene related to a syndrome resembling human premature aging, functions in a negative regulatory circuit of vitamin D endocrine system. Mol. Endocrinol. 17, 2393–2403 (2003). 14528024 10.1210/me.2003-0048

[b31] Ohnishi M., Nakatani T., Lanske B. & Razzaque M. S. Reversal of mineral ion homeostasis and soft-tissue calcification of klotho knockout mice by deletion of vitamin D 1α-hydroxylase. Kidney Int. 75, 1166–1172 (2009). 19225558 10.1038/ki.2009.24 PMC3143194

[b32] Manya H. *et al.* Klotho protein deficiency leads to overactivation of μ-Calpain. J. Biol. Chem. 277, 35503–35508 (2002). 12119304 10.1074/jbc.M206033200

[b33] Koh N. *et al.* Severely reduced production of klotho in human chronic renal failure kidney. Biochem. Biophys. Res. Commun. 280, 1015–1020 (2001). 11162628 10.1006/bbrc.2000.4226

[b34] Hu M.-C. & Moe O. W. Klotho as a potential biomarker and therapy for acute kidney injury. Nat. Rev. Nephrol. 8, 423–429 (2012). 22664739 10.1038/nrneph.2012.92 PMC3752896

[b35] Panesso M. C. *et al.* Klotho has dual protective effects on cisplatin-induced acute kidney injury. Kidney Int. 85, 855–870 (2014). 24304882 10.1038/ki.2013.489 PMC3972320

[b36] Tsai S. *et al.* Mouse development with a single E2F activator. Nature 454, 71137–71141 (2008). 10.1038/nature07066 PMC4288824 18594513

[b37] Kawano K. *et al.* Klotho gene polymorphisms associated with bone density of aged postmenopausal women. J. Bone Miner. Res. 17, 1744–1751 (2002). 12369777 10.1359/jbmr.2002.17.10.1744

[b38] Chkhotua A. B. *et al.* Increased expression of p16^INK4a^ and p27^Kip1^ cyclin-dependent kinase inhibitor genes in aging human kidney and chronic allograft nephropathy. Am. J. Kidney Dis. 41, 1303–1313 (2003). 12776284 10.1016/s0272-6386(03)00363-9

[b39] Melk A. *et al.* Expression of p16^INK4a^ and other cell cycle regulator and senescence associated genes in aging human kidney. Kidney Int. 65, 510–520 (2004). 14717921 10.1111/j.1523-1755.2004.00438.x

[b40] Semba R. D. *et al.* Plasma klotho and mortality risk in older community- dwelling adults. J. Gerontol. 66A, 794–800 (2011). 10.1093/gerona/glr058 PMC3143348 21474560

[b41] Vijg J. & Campisi J. Puzzles, promises and a cure for ageing. Nature 454, 1065–1071 (2008). 18756247 10.1038/nature07216 PMC2774752

[b42] Arking D. E. *et al.* Association of human aging with a functional variant of klotho. Proc. Natl Acad. Sci. USA 99, 856–861 (2002). 11792841 10.1073/pnas.022484299 PMC117395

[b43] Shimoyama Y., Nishio K., Hamajima N. & Niwa T. Klotho gene polymorphisms G-395 A and C1818T are associated with lipid and glucose metabolism, bone mineral density and systolic blood pressure in Japanese healthy subjects. Clin. Chim. Acta 406, 134–138 (2009). 19539617 10.1016/j.cca.2009.06.011

[b44] Beausejour C. M. & Campisi J. Aging: balancing regeneration and cancer. Nature 443, 404–405 (2006). 16957734 10.1038/nature05221

[b45] Sharpless N. E. *et al.* Loss of p16^Ink4a^ with retention of p19^Arf^ predisposes mice to tumorigenesis. Nature 416, 86–91 (2001). 11544531 10.1038/35092592

[b46] Yoshimoto S. *et al.* Obesity-induced gut microbial metabolite promotes liver cancer through senescence secretome. Nature 499, 97–101 (2013). 23803760 10.1038/nature12347

[b47] Cho E. A. *et al.* Differential expression and function of cadherin-6 during renal epithelium development. Development 125, 803–812 (1998). 9449663 10.1242/dev.125.5.803

[b48] Esteban M. A. *et al.* Regulation of E-cadherin expression by *VHL* and hypoxia-induced factor. Cancer Res. 66, 3567–3575 (2006). 16585181 10.1158/0008-5472.CAN-05-2670

[b49] Prozialeck W. C., Lamar P. C. & Appelt D. M. Differential expression of E-cadherin, N-cadherin and beta-catenin in proximal and distal segments of the rat nephron. BMC Physiol. 4, 10 (2004). 15147582 10.1186/1472-6793-4-10 PMC459230

[b50] Tomas-Loba A. *et al.* Telomerase reverse transcriptase delays aging in cancer-resistant mice. Cell 135, 609–622 (2008). 19013273 10.1016/j.cell.2008.09.034

[b51] Vlahovic G., Russell M. L., Mercer R. R. & Carapo J. D. Cellular and connective tissue changes in alveolar septal walls in emphysema. Am. J. Respir. Crit. Care Med. 160, 2086–2092 (1999). 10588633 10.1164/ajrccm.160.6.9706031

[b52] Imai Y. *et al.* Cross-talk between the Rb pathway and AKT signalling forms a quiescence-senescence switch. Cell Rep. 7, 194–207 (2014). 24703840 10.1016/j.celrep.2014.03.006

[b53] Takahashi A. *et al.* DNA damage signaling triggers degradation of histone methyl-transferases through APC/C^Cdh1^ in senescent cells. Mol. Cell 45, 123–131 (2012). 22178396 10.1016/j.molcel.2011.10.018

[b54] Takeuchi S. *et al.* Intrinsic cooperation between p16^INK4a^ and p21^Waf1/Cip1^ in the onset of cellular senescence and tumor suppression *in vivo*. Cancer Res. 70, 9381–9390 (2010). 21062974 10.1158/0008-5472.CAN-10-0801

