# Supplementary material for: Ablation of the p16INK4a tumour suppressor reverses ageing phenotypes of klotho mice
Source: Nat Commun. 2015 Apr 29;6:7035. doi: 10.1038/ncomms8035 (PMC4421814; doi:10.1038/ncomms8035)
Supplement: Supplementary Information — Supplementary Figures 1-11 and Supplementary Table 1 [file ncomms8035-s1.pdf]

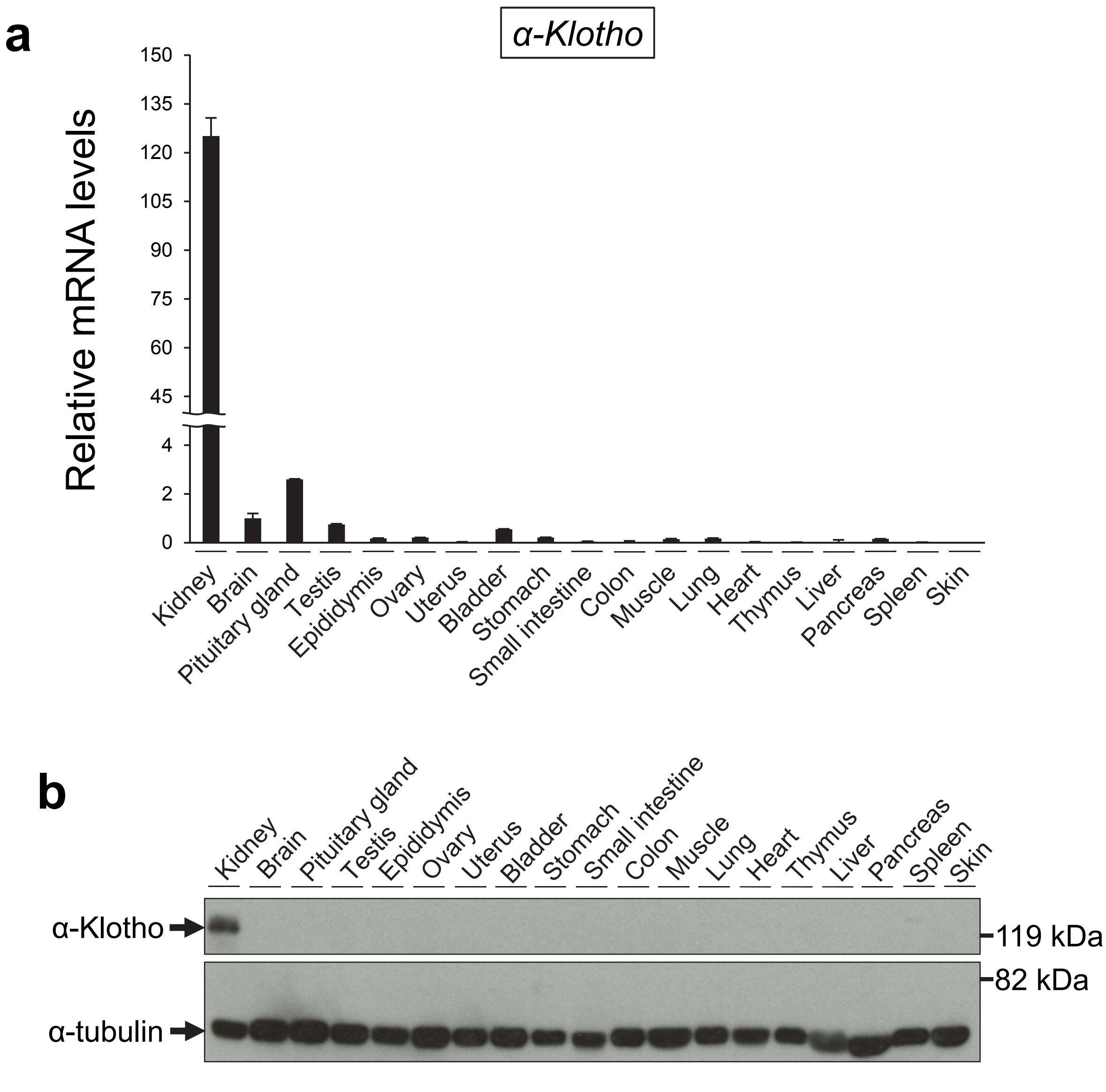

Supplementary Figure 1. Tissue distribution of *α-klotho*.

(a) The relative expression levels of *α-klotho* mRNA in the indicated tissue of *WT* mice were determined by RT-qPCR. All samples except those from the ovary and uterus were collected from 16-week-old male mice. Samples from ovary and uterus were collected from 16-week-old female mice. The experiments were performed in triplicate using three individual mice, and representative results from three independent experiments are shown. Error bars indicate mean ± s.d.

(b) Indicated tissue of 16-week-old female (ovary and uterus) and male (other tissues) *WT* mice were subjected to western blot analysis using antibodies shown left. α-tubulin was used as a loading control. The experiments were performed in triplicate using three individual mice, and representative results from three independent experiments are shown.

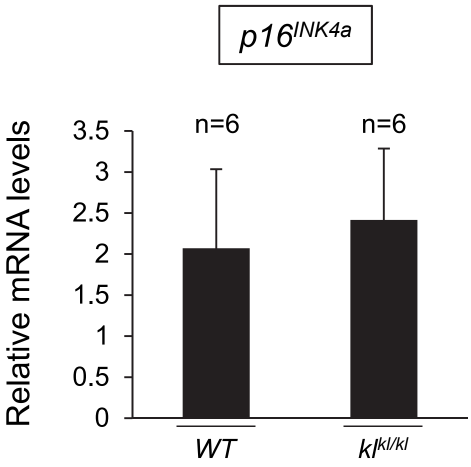

Supplementary Figure 2. Renal expression of *p16*^{INK4a} in *kl*$^{-/-}$ mice.
The relative levels of *p16*^{INK4a} mRNA in kidneys of 2 to 6 month-old male mice of each genotype were examined by RT-qPCR. n=number of mice examined. Data were analysed by Student's *t*-test and are displayed as mean ± s.d.

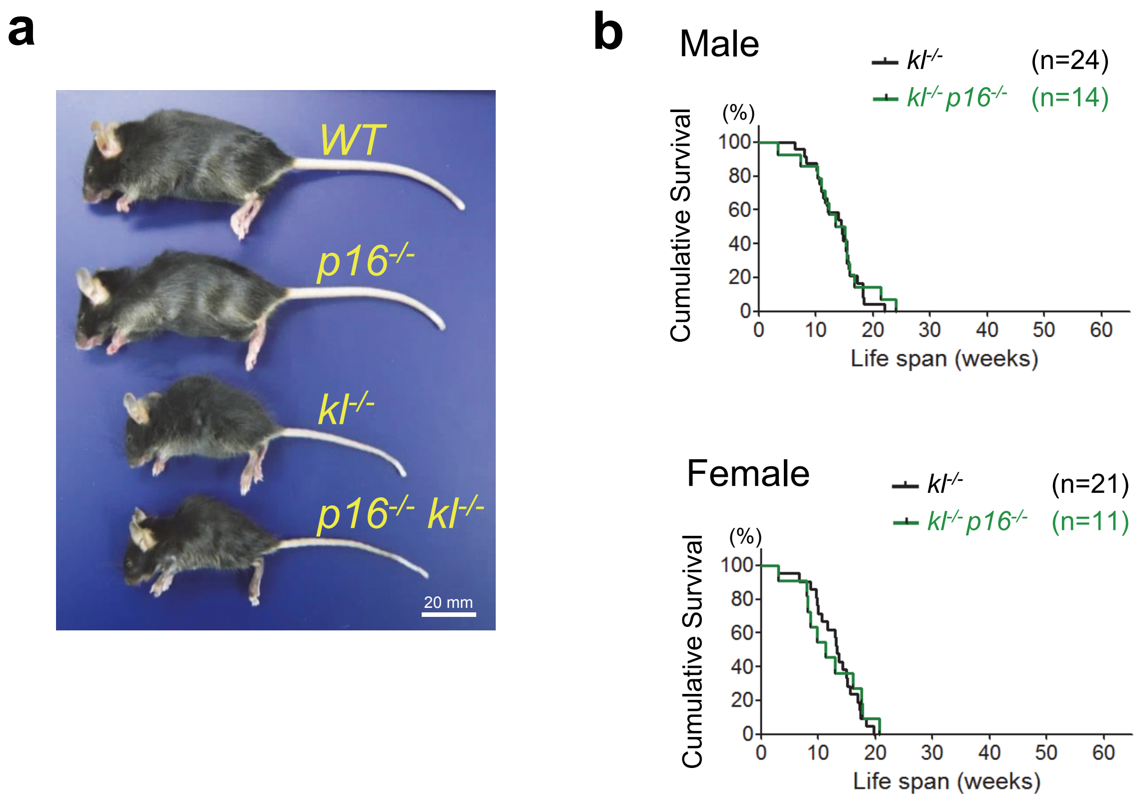

Supplementary Figure 3. ***p16**^{INK4a}* **ablation failed to reverse the ageing phenotypes of *kl*$^{-/-}$ mice. (a)** Representative photographs of 11-week-old female mice of each genotype (n=3). **(b)** Kaplan-Meier plot showing survival of *kl*$^{-/-}$ mice (male, n=24; female, n=21), and *p16*$^{-/-}$ *kl*$^{-/-}$ mice (male, n=14 ; female, n= 11).

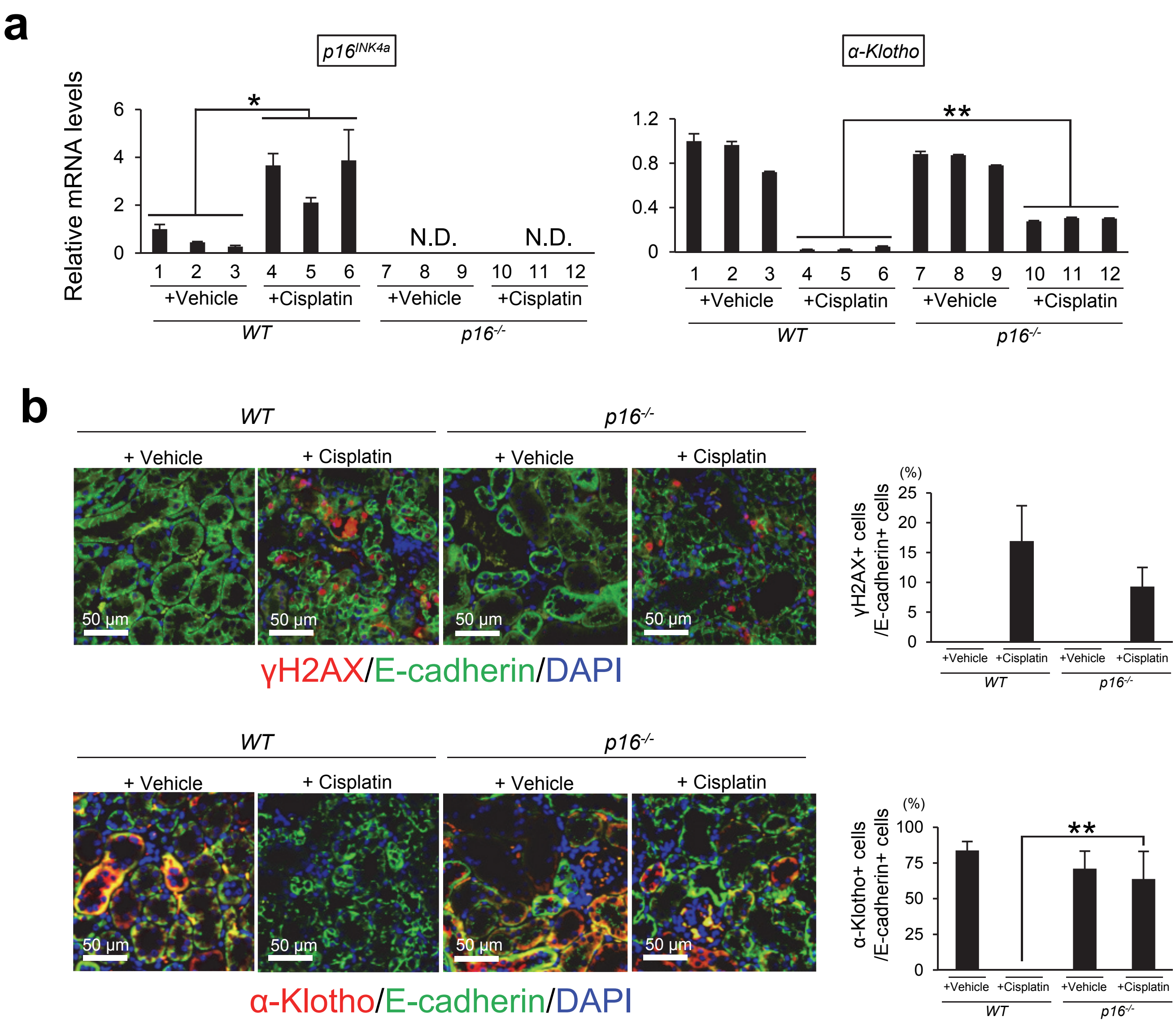

Supplementary Figure 4. ***p16^{INK4a}*** **ablation mitigates Cisplatin-induced reduction of** ***α-klotho*****.**

(a) The relative levels of indicated mRNA in kidneys of 10 to 20-week-old male mice of each genotype treated with or without Cisplatin were examined by RT-qPCR. Representative results of three individual male mice were shown (n=3 experiments). **(b)** Immunohistochemical analysis of γ-H2AX (red), E-cadherin (green), and DAPI (blue) (upper panel) and α-klotho (red), E-cadherin (green), and DAPI (blue) (lower panel) in kidney sections of 10 to 20-week-old male mice of each genotype treated with or without Cisplatin. Representative results of three individual male mice were shown. The histograms indicate the percentages of E-cadherin expressing cells that were positive for γ-H2AX (upper panel) or α-klotho expression (lower panel). At least 100 cells were scored per group (n=3 experiments). For all graphs, data were analysed by Welch's *t*-test and are displayed as mean ± s.d. *P<0.05 **P<0.01.

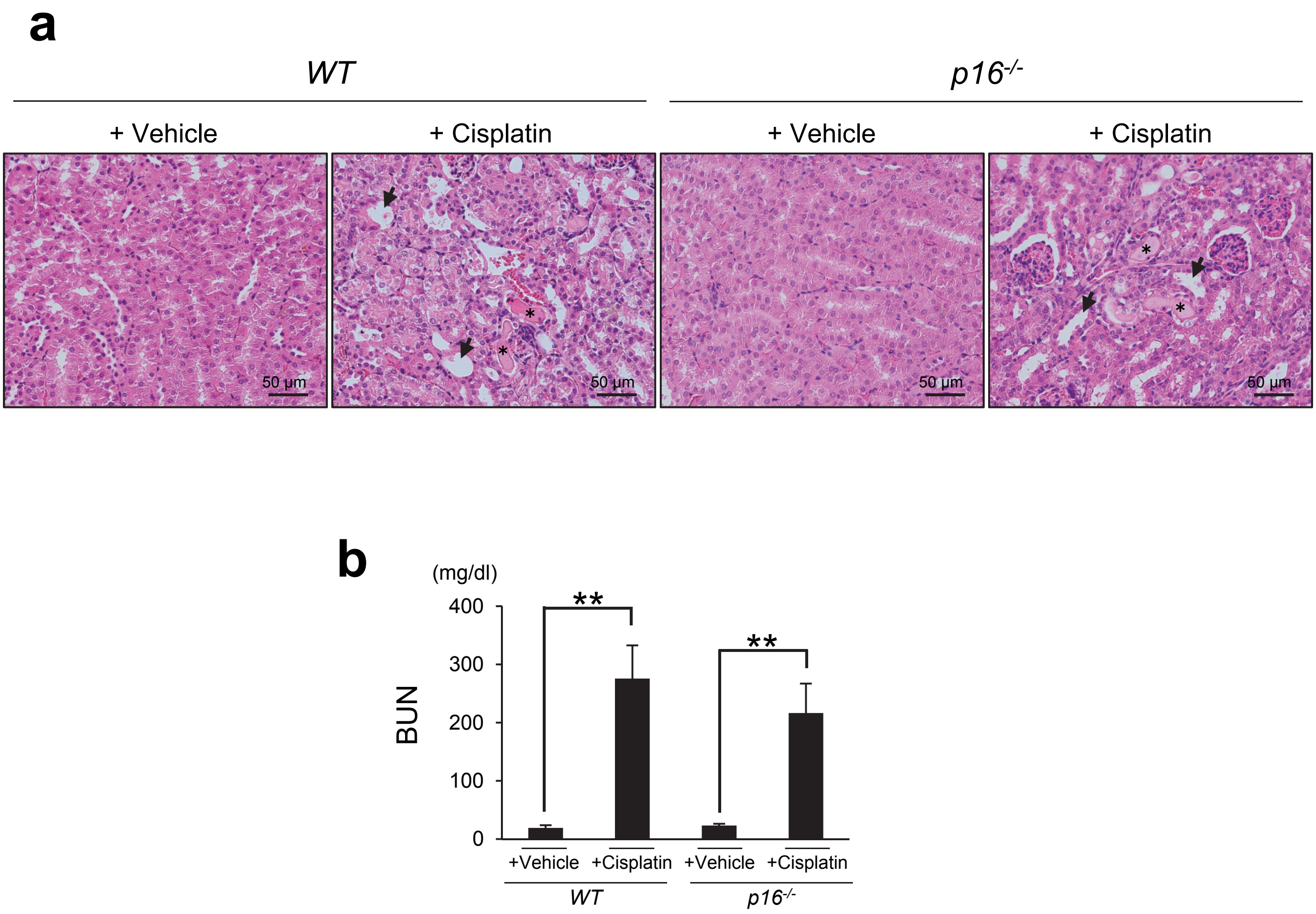

Supplementary Figure 5. Cisplatin-induced acute kidney injury.
(a) Representative HE staining of kidney sections from 10 to 20-week-old *WT* and *p16*$^{-/-}$ male mice treated with or without cisplatin (see supplementary Figure 4) (n=3). An asterisk represents renal tubular casts and an arrow represents dilated renal tubules. (**b)** Serum levels of blood urea nitrogen (BUN) were measured after treatment with cisplatin three times (n=3). Data were analysed by Student's *t*-test and are displayed as mean ± s.d. $^{**}P<0.01$.

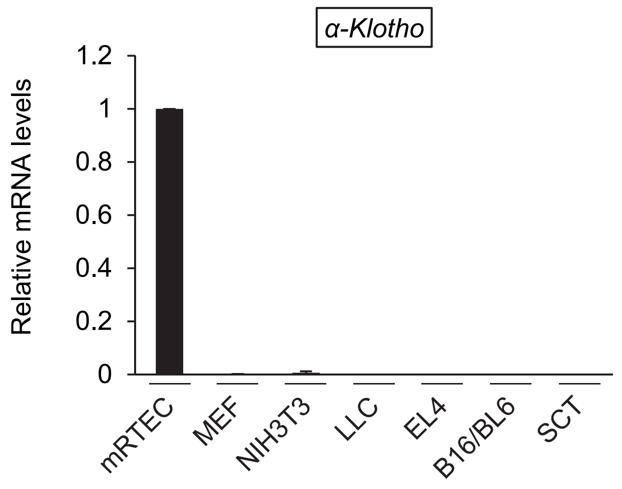

Supplementary Figure 6. Comparison of *α-klotho* expression among murine cells. Relative expression levels of *α-klotho* mRNA in the indicated murine cells were determined by RT-qPCR. SCT: Spindle cell tumour cell line prepared from mice lacking $p16^{INK4a}$ genes[54]. The experiments were performed in triplicate, and representative results from three independent experiments are shown. Error bars indicate mean ± s.d.

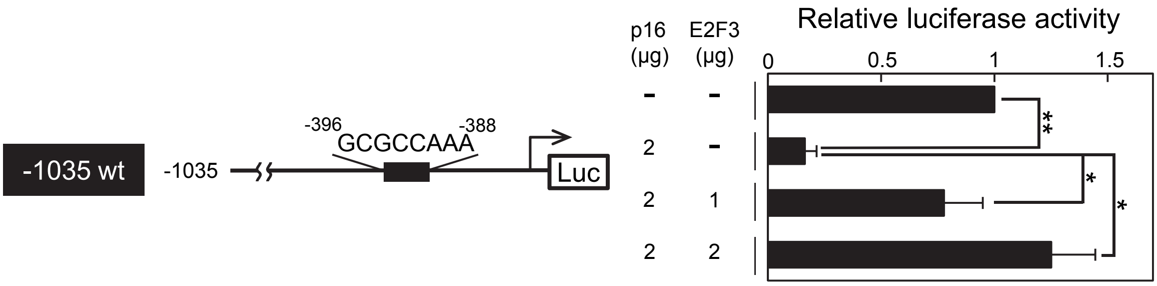

Supplementary Figure 7. Ectopic expression of E2F3 blunts negative effect of p16^{INK4a}. Schematic representation of the reporter construct of mouse *α-klotho* gene promoter used in the analysis (left panel). The E2F binding element is shown as a black rectangle with the sequence and firefly luciferase is shown as Luc. The reporter construct was co-transfected into mRTECs along with LacZ plasmid. Where indicated, cells were also co-transfected with a p16^{INK4a} expression plasmid and increasing amounts of E2F3 expression plasmid. The experiments were performed in triplicate, and representative results from three independent experiments are shown. Data were analysed by Welch's *t*-test and are displayed as mean ± s.d. *P<0.05 **P<0.01.

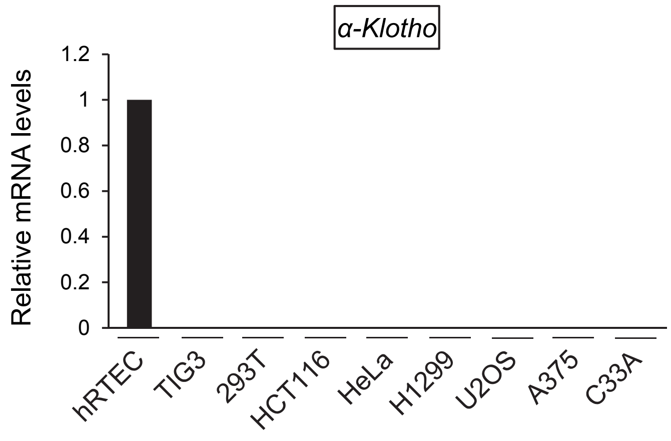

Supplementary Figure 8. Comparison of *α-klotho* expression among human cells. Relative expression levels of *α-klotho* mRNA in the indicated human cells were determined by RT-qPCR. The experiments were performed in triplicate, and representative results from three independent experiments are shown. Error bars indicate mean ± s.d.

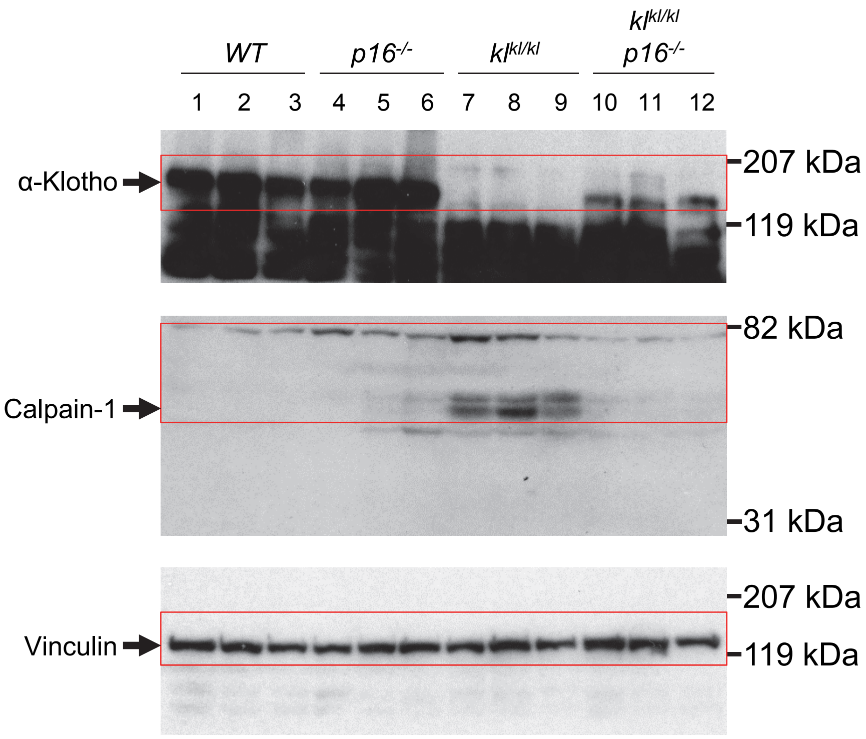

Supplementary Figure 9. Expanded blots from Figure 4c.

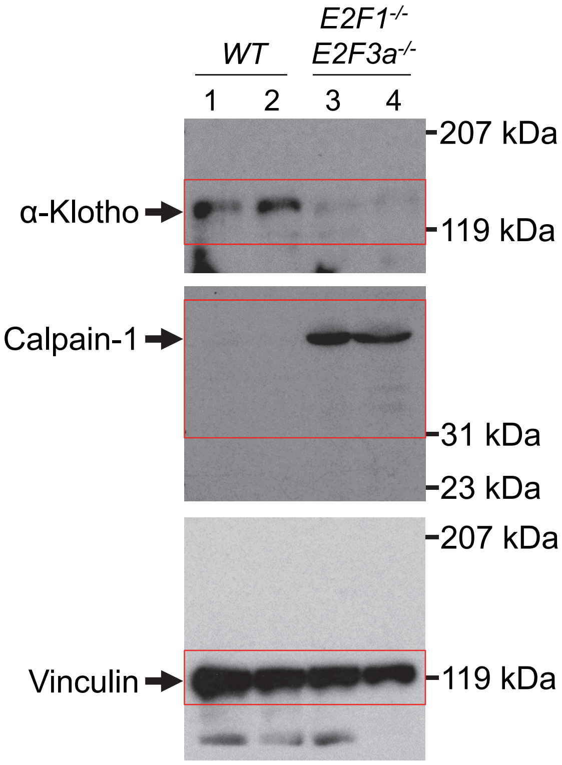

Supplementary Figure 10. Expanded blots from Figure 6d.

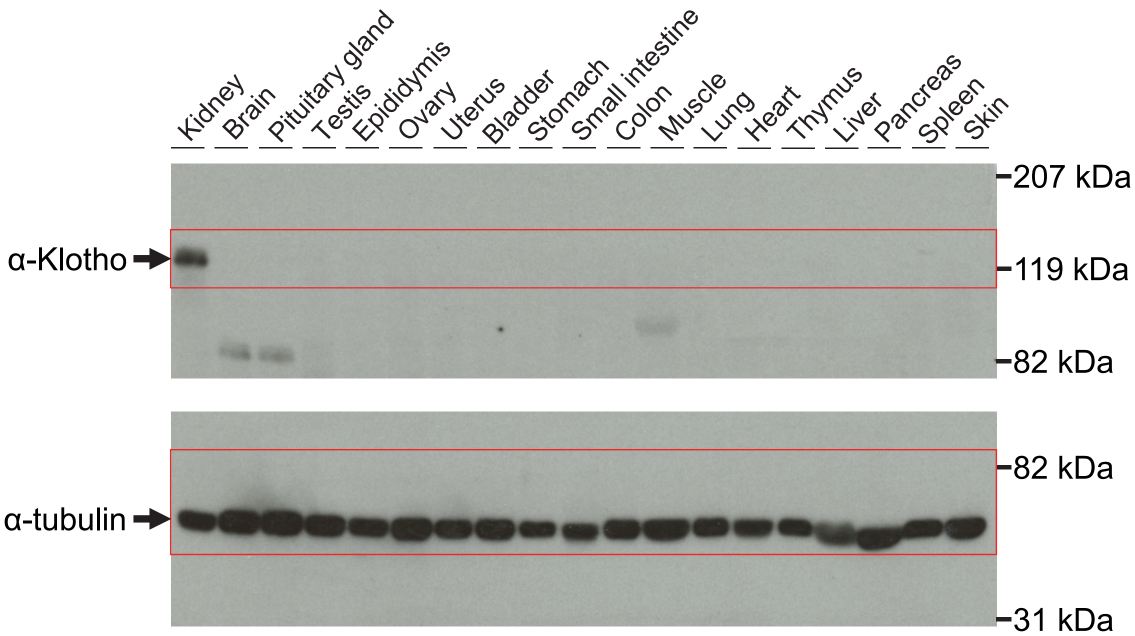

Supplementary Figure 11. Expanded blots from Supplementary Figure 1b.

Supplementary Table 1. Primers used for RT-qPCR.

Target gene	Sequence	
mouse GAPDH	5'-CAACTACATGGTCTACATGTTC-3'<br>5'-CGCCAGTAGACTCCACGAC-3'	(forward)<br>(reverse)
mouse α-klotho	5'-GATGGCAGAGAAATCAACACAGT-3'<br>5'-ACTACGTTCAAGTGGACACTACT-3'	(forward-1)<br>(reverse-1)
mouse α-klotho	5'-CTGAAAACCAGCCCCTTGAA-3'<br>5'-AATAAGCCTCTTACTGTGAT-3'	(forward-2)<br>(reverse-2)
mouse p16	5'-AGGGCCGTGTGCATGACGTG-3'<br>5'-GCACCGGGCGGGAGAAGGTA-3'	(forward-1)<br>(reverse-1)
mouse p16	5'- GAACTCTTTCGGTCGTACCC-3'<br>5'- CGAATCTGCACCGTAGTTGA-3'	(forward-2)<br>(reverse-2)
mouse Cyp27b1	5'-TGCTTGCGGATTGCTAACG-3'<br>5'-CCTTAGTCGTCGCACAAGGTC-3	(forward)<br>(reverse)
human GAPDH	5'-CAACTACATGGTTTACATGTTC-3'<br>5'-GCCAGTGGACTCCACGAC-3'	(forward)<br>(reverse)
human α-klotho	5'-TACCTGGTGGCGCACAACCT-3'<br>5'-TGTGGTCGGTCATTCTTCGA-3'	(forward)<br>(reverse)
human p16	5'-CGAATAGTTACGGTCGGAGG-3'<br>5'-TGAGAGTGGCGGGGTCG-3'	(forward)<br>(reverse)
human Cdc6	5'-GGCACAGGCTACAATCAGTT-3'<br>5'-AATCTCCTGAGCAATAGCTG-3'	(forward)<br>(reverse)
